# Supplementary material for: Improving Outcomes in the Advanced Gastrointestinal Stromal Tumors: The Role of the Multidisciplinary Team Discussion Intervention
Source: J Pers Med. 2023 Feb 26;13(3):417. doi: 10.3390/jpm13030417 (PMC10057951; doi:10.3390/jpm13030417)
Supplement: Supplementary file 1 [file jpm-13-00417-s001.zip › jpm-2221510-supp.pdf]

GIST MDT Discussion

March * *, 2019

The results of Kit gene mutation

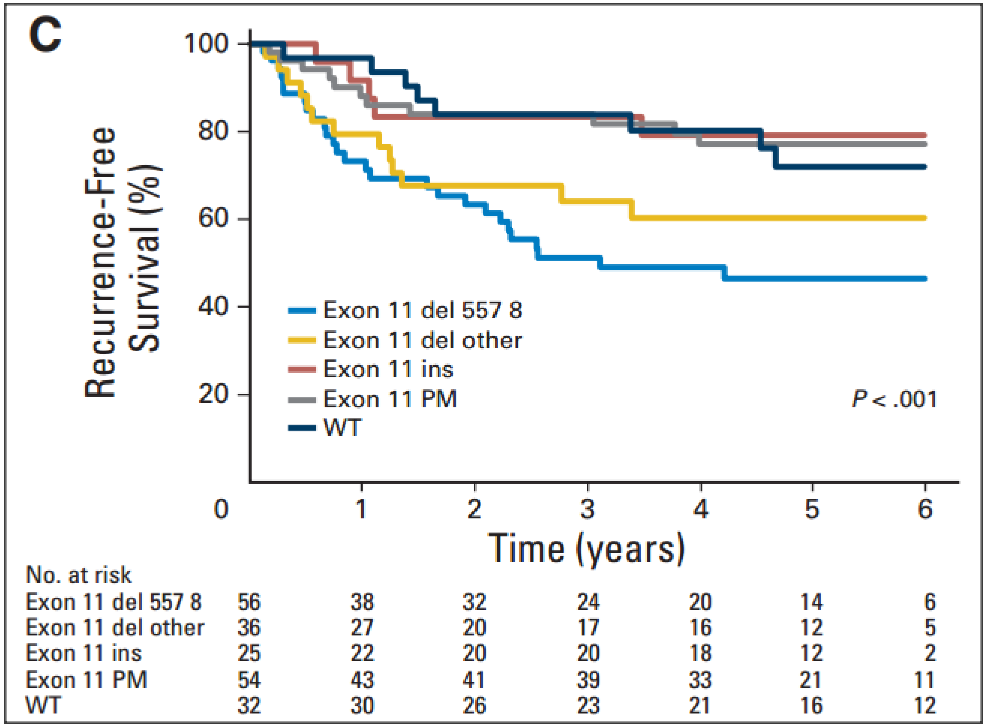

The Kit11 L576P mutation is primary resistant to IM

The primary Kit13 K642E mutation is sensitive to IM

Secondary Kit13 V654A was resistant to IM

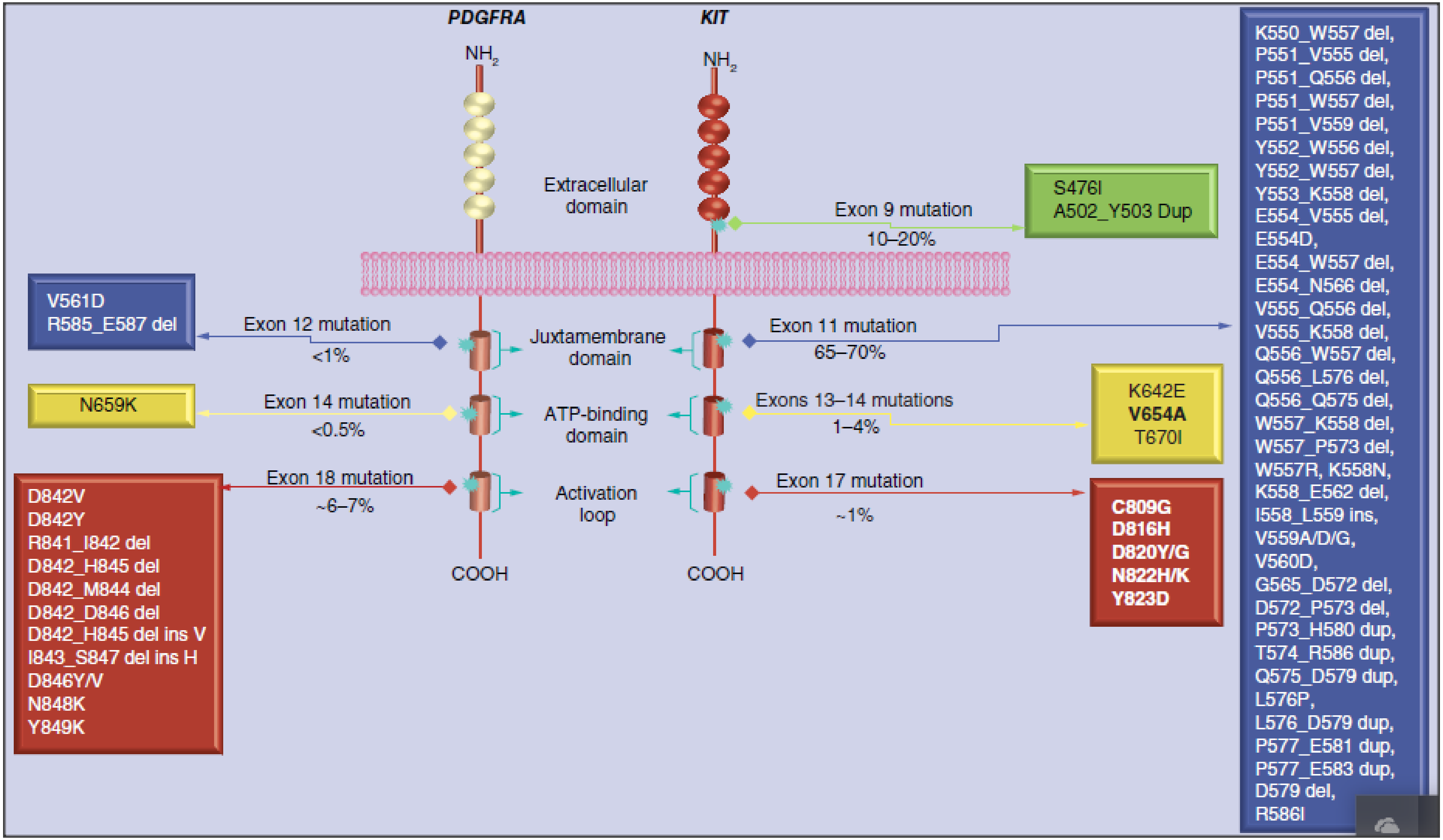

PDGFRA
KIT
NH2
NH2
Extracellular domain
Exon 9 mutation
10–20%
S476I
A502_Y503 Dup
V561D
R585_E587 del
Exon 12 mutation
<1%
Juxtamembrane domain
Exon 11 mutation
65–70%
N659K
Exon 14 mutation
<0.5%
ATP-binding domain
Exons 13–14 mutations
1–4%
K642E
V654A
T670I
Exon 18 mutation
~6–7%
Activation loop
Exon 17 mutation
~1%
D842V
D842Y
R841_I842 del
D842_H845 del
D842_M844 del
D842_D846 del
D842_H845 del ins V
I843_S847 del ins H
D846Y/V
N848K
Y849K
C809G
D816H
D820Y/G
N822H/K
Y823D
COOH
COOH
K550_W557 del,
P551_V555 del,
P551_Q556 del,
P551_W557 del,
P551_V559 del,
Y552_W556 del,
Y552_W557 del,
Y553_K558 del,
E554_V555 del,
E554D,
E554_W557 del,
E554_N566 del,
V555_Q556 del,
V555_K558 del,
Q556_W557 del,
Q556_L576 del,
Q556_Q575 del,
W557_K558 del,
W557_P573 del,
W557R, K558N,
K558_E562 del,
I558_L559 ins,
V559A/D/G,
V560D,
G565_D572 del,
D572_P573 del,
P573_H580 dup,
T574_R586 dup,
Q575_D579 dup,
L576P,
L576_D579 dup,
P577_E581 dup,
P577_E583 dup,
D579 del,
R586I

The first patient

Basic Information

Wang * Female 56 years of age

- In March 2018, “Transanorectal mass resection " was performed in a hospital. Intraoperative results showed that the tumor was completely removed by an electric knife at 5 o 'clock at K-C position with solid nodules of about 2cm in size at about 3cm from the anal margin.
- Postoperative examination: rectal GIST, with a maximum diameter of 3cm and mitotic count of 3/ 50HPF.
- Immunohistochemistry: DOG-1 (+), CD117 (+), CD34 (+).
- Gene detection: Ins502-503 point mutation was observed in C-KIT 9.
- Start taking IM 400mg/ day after surgery.

Review of medical history

She was diagnosed with rectal GIST in a hospital in March 2018.

Blood drug concentration：

2244.18ng/ml（2018.7.3）；
2593.39ng/ml（2018.8.28）；
1102.58ng/ml（2018.10.30）。

The CT report on August 29, 2018:1. In the enhanced scan, the lamp-like enhancement shadow appeared in the lower rectum. Considering the possibility of hemorrhoid vein dilation, the enhancement degree of CT was less than that on March 5, 2018. 2. Slightly low-density nodules under the diaphragmatic surface of the upper right posterior lobe of the liver were enhanced in the posterior vein, which was a new occurrence compared with the previous one. Considering the possibility of metastasis, further examination was recommended. A few cysts in the lower segment of the right hepatic lobe.

Review of medical history

On November 25, 2018, the liver Primovist MRI display showed that the S2 and S7 segments of the liver were mildly enhanced, and the liver located in the S2 segment was larger, about 1.3*1.0cm, which considered the possibility of metastasis.

Blood drug concentration：
1882.18ng/ml（2019.1.22）；
1809.36ng/ml（2019.2.26）

2018-12-18 MDT： 1. Abdominal CT+ Cancer spectrum examination was performed；
2. It is recommended to increase the dosage of IM to 600mg/d and re-examine the patient to evaluate whether there are indications for surgery 2-3 months later.

On December 22, 2018, the dosage of IM was increased to 600mg/d.

2018-12-20 CT：

Nodules in the S2 segment (0.9cm) and S7 segment (0.6cm) of the liver are compared with old MRI images taken on November 25, 2018, suggesting that nodules in the S7 segment of the liver are slightly larger than before, indicating possible metastasis.

2019-03-04 CT：

1、Nodules in S2 segment of liver (about 1.3*1.0cm in size);

2、There were slightly low-density nodules in the S7 segment of the liver, and the results were similar to the previous examination;

- MDT: Curative effect? The next step?
- MDT Discussion Intervention:

S2 segment of the liver: Hemangioma? Metastatic tumors? From 1.2cm to 1.4cm.

S7 segment of the liver: New lesion with no obvious progression.

It is recommended to review B-ultrasound to clarify the lesion nature of the S2 segment of the liver.

The second patient

Basic Information

Mou **， Male，67 years of age

In December 2004, he received "proximal subtotal gastrectomy + splenectomy" in a hospital.

Postoperative pathological examination indicated that gastric GIST.

No drugs were taken for adjuvant therapy after surgery

Review of medical history

In July 2016, abdominal tumor resection and partial transverse colon resection were performed.
A genetic test was performed in November 2017, and the result showed C-KIT11 (c.1665-1688del).

Kit11 mutation (P.Gln556-Il563del)

In October 2009, the left liver, caudal lobe of liver and transverse mesentery were resected in a hospital

The CT report in November 2017 showed the possibility of multiple cysts in the liver. The larger one was located in the S4 segment of the liver, about 1.0*1.6cm in size. After enhancement, the lesions showed no enhancement. The splenic region is a mass of flaky shadow, and there are scattered small flaky enhanced shadows, which is the possibility of encapsulated effusion.

November 2009 - November 2014
Sutent 50mg

December 2016 - present
IM 400mg

Review of medical history

2017.11 MDT Discussion: Before the operation in the other hospital, the lesion of the spleen and fossa had been shown in the preoperative CT examination, so far no progression of the lesion was observed, and the liver lesion showed no significant change compared with the previous.

1. Abdominal B-ultrasound was performed once a month to follow up on the lesions in the spleen and liver;
2. The remaining treatment plan remains unchanged.

2018-11-20 MDT Discussion:

Suggestions:

1. Complete the puncture biopsy of spleen lesions and perform gene detection.
2. The patient refused to change the drug, so the daily 400mg IM treatment was continued, and the next step of drug adjustment was made after the genetic test results were indicated.
3. Liver CT examination suggested that the liver lesion might be a cyst, so it was necessary to require an MRI examination of the liver.

- MDT：　What about the lesions? The next step?
- The CT examination on March 19, 2019, compared with the previous examination, the liver lesions were relatively stable (appeared to progress;
- The patient maintained IM at 400mg/ day;
- Suggestion: B ultrasound/month.

The third patient

Basic Information

Zhang **， Male，67 years of age

- In July 2015, she was admitted to the hospital for "right upper abdominal pain for 4+ years". CT examination was performed, and the results showed that there was a mass in the left lower abdomen. There are two circular lesions in the right lobe of the liver, considering the possibility of tumor metastasis. Abdominal exploration was performed and small intestinal tumor resection was performed. Because the liver was closed by the greater omentum adhesion, the liver lesion could not be explored in this operation;
- Postoperative examination: small intestine GIST, the maximum diameter of tumor was 6cm, mitotic count > 10/50HPF, liver lesion resection was not performed;
- Immunohistochemistry: DOG-1 (+), CD117 (+), CD34 (-);
- Gene detection: V559D mutation occurred in the region detected by the C-KIT exon-11 gene.

Review of medical history

In August 2015, small intestinal GIST resection was performed.

B-ultrasound results of our hospital in March 2017 showed:

1. Abnormal hypoechoic in the liver, about 13*8mm in size;
2. There are space-occupying lesions in the liver.

The CT report in September 2016 showed that there were multiple circular low-density nodules in the liver, the largest of which was about 27mm in diameter.

The CT report in February 2018 showed that there were multiple circular low-density nodules in the liver, the largest of which was about 27mm in diameter. Considering the possibility of metastasis, compared with the previous CT (2016-09), the size of the lesions did not change significantly, but the number of lesions increased.

September 2015 - present
IM 400mg

2018-03MDT：

2018-02 CT： Progression of liver lesions.

Recommendations:

1. Hepatic lesion intervention + radiofrequency ablation (Only a part of the lesions can be resolved);

2. Take IM regularly and review the blood concentration regularly.

2018-09 B ultrasound：

1. There is abnormal hypoechoic in the liver (metastasis is possible), the larger one is about 30*26mm, located in the upper segment of the right posterior lobe of the liver (S7 segment), and there is a non-echo area of about 22*18mm in the liver.

2. There was a mixed space-occupying lesion in the liver, about 9*7mm in size, located in the lower segment of the left external lobe of the liver (S3 segment); Compared with the previous examination (2017-03-09), there were no significant changes in the lesions.

- 2019-02 CT:

1. The small intestine wall of the right mid-abdomen was slightly thickened with a strip of high-density shadow, which was considered a postoperative change;

2. Ascending colon thickening;

3. Multiple nodules in the liver were considered as metastases. The number of lesions was slightly more than before (2018-02-06), and the largest lesion diameter was about 2.7cm.

Blood drug concentration: 583.14ng/ml (2017.4.11);

1543.28ng/ml (2017.7.5);

1502.94ng/ml (2018.4.10);

2025.22ng/ml (2018.9.4).

- MDT: Progression of metastatic lesions? The next step?

MDT Discussion Intervention:

Progression of liver lesions (more than 10 scattered lesions), replacement of targeted drugs.
